# Transcobalamin receptor CD320, responsible for vitamin B_12_ cellular uptake, is present on the cell surface as a homo-oligomer

**DOI:** 10.1016/j.jbc.2026.113286

**Published:** 2026-07-16

**Authors:** Wenjun Guo, Renping Qiu, Xiaotong Zhao, Tiantian Zhou, Meng Liu, Ningzheng Dong, Qingyu Wu

**Affiliations:** 1 NHC Key Laboratory of Thrombosis and Hemostasis, Jiangsu Institute of Hematology, The First Affiliated Hospital of Soochow University, Soochow University Suzhou Medical College, Suzhou, China; 2 Cyrus Tang Hematology Center, Collaborative Innovation Center of Hematology, State Key Laboratory of Radiation Medicine and Prevention, Soochow University, Suzhou, China

**Keywords:** CD320, cell surface receptor, cobalamin, oligomerization, vitamin B_12_ metabolism

## Abstract

CD320, also known as the transcobalamin receptor, is a key receptor that mediates the cellular uptake of circulating vitamin B_12_ bound to transcobalamin. In humans, CD320 abnormalities cause metabolic and neurological disorders. Previous biochemical studies indicate that the CD320 is a monomeric receptor. In this study, we conducted molecular and cellular experiments in human embryonic kidney 293 cells to examine biosynthesis and molecular forms of human CD320. We found that CD320 was present on the cell surface in an oligomeric form, which consisted of five homomers interconnected *via* disulfide bonds, possibly in one or both low-density lipoprotein receptor (LDLR)-like domains. Deletion of the LDLR-like domains prevented CD320 oligomerization and cell surface localization, thereby impairing cellular uptake of vitamin B_12_. By analyzing biochemical forms of CD320 in intracellular compartments, we showed that CD320 oligomerization occurred in the endoplasmic reticulum (ER) and that this process required the transmembrane domain but not N- or O-glycosylation of CD320. Moreover, we showed that the CD320 ΔE88 variant, identified in infants with abnormal vitamin B_12_ metabolism, did not prevent CD320 oligomerization but delayed CD320 trafficking out of the ER, resulting in low levels of CD320 oligomers on the cell surface. Together, our findings provide important insights into the biochemical nature and cellular mechanisms underlying the function of CD320 and associated pathologies in vitamin B_12_ metabolism.

Vitamin B_12_, also known as cobalamin, plays an essential role in DNA synthesis, fatty acid and protein metabolism, blood cell production, and neurological function by acting as a cofactor for methionine synthase in the cytosol and methylmalonyl coenzyme A mutase in the mitochondria (1, 2, 3). Inadequate dietary intake or malabsorption may result in vitamin B_12_ deficiency, leading to severe conditions such as megaloblastic anemia and demyelinating neurologic disorders (4, 5, 6). Recent studies have shown that inhibition of cellular uptake of vitamin B_12_ in the central nervous system by autoantibodies is another pathological mechanism in patients with central nervous system-related conditions (*e*.*g*., ataxia, cognitive decline, and memory loss) (7, 8, 9).

Vitamin B_12_ uptake relies on multiple cellular receptors in diverse tissues (3, 10). In the small intestine, vitamin B_12_ absorption is mediated by cubam (formerly known as cubilin) on the ileal apical membrane. In the kidney, vitamin B_12_ reabsorption is carried out by megalin on the luminal surface of the proximal tubules. The cellular uptake of B_12_, circulating in blood in complex with transcobalamin (*i*.*e*., holo-TC), is mediated primarily *via* CD320 (also known as the transcobalamin receptor) (3, 10, 11, 12), a type I transmembrane protein consisting of an N-terminal signal peptide, an extracellular region with two low-density lipoprotein receptor class A (LDLR)-like domains separated by an epidermal growth factor (EGF)-like module, a single-span transmembrane (TM) domain, and a C-terminal cytoplasmic tail (13). The LDLR-like domains mediate the holo-TC binding, whereas the cytoplasmic segment participates in the receptor-mediated endocytosis (14, 15). It has been shown that the human LDLR may also participate in the cellular uptake of holo-TC in peripheral tissues, which serves as a compensatory mechanism in response to CD320 deficiency (7).

CD320 is expressed in diverse human tissues. Based on the Human Protein Atlas database (16, 17), CD320 mRNA and protein are found in fibroblasts and epithelial, endothelial and proliferating cells in a wide range of normal tissues. In polarized cells, for example, luminal epithelial cells in the small intestine and proximal tubular epithelial cells in the kidney, CD320 is expressed on the apical cell surface, suggesting a potential role of CD320 in vitamin B_12_ absorption and reabsorption (18, 19). In cancers, including hepatocellular carcinoma, breast and gastric cancers, osteosarcoma and multiple myeloma, increased CD320 expression is associated with enhanced DNA synthesis and cell proliferation (20, 21, 22, 23, 24, 25, 26, 27). Targeting CD320 has been proposed as a potential anti-cancer strategy (23, 24, 25, 28). These data highlight an important role of CD320 in human pathophysiology.

Despite advances in understanding the role of CD320 in vitamin B_12_ metabolism and related neurological disorders, molecular and cellular mechanisms in regulating CD320 biosynthesis and function are not fully understood. In polarized renal and intestinal epithelial cells, an amino acid signal in the second LDLR-like domain has been identified, which may act in a Rab11a-dependent mechanism directing CD320 to the apical cell membrane (18, 19). Previous studies indicated that CD320 functions as a monomeric receptor (15, 29, 30). In a recent study, however, we found that a complex form of CD320 may exist on the cell surface, although the biological significance of this observation remained unknown (31).

To verify the biochemical form of CD320 on the cell surface and to understand biochemical and cellular mechanisms underlying CD320 complex formation, we examined CD320 subcellular distribution and protein forms in human embryonic kidney 293 (HEK293) cells. Moreover, we analyzed CD320 mutants lacking individual protein modules and a naturally occurring CD320 variant identified in patients with impaired vitamin B_12_ uptake. The results from our experiments reveal important insights into the oligomeric nature of CD320, which should help elucidate the biochemical and cellular mechanisms underlying the function of CD320 in vitamin B_12_ metabolism and associated disorders.

## Results

### CD320 is present as oligomers on the cell surface

**Figure 1:**
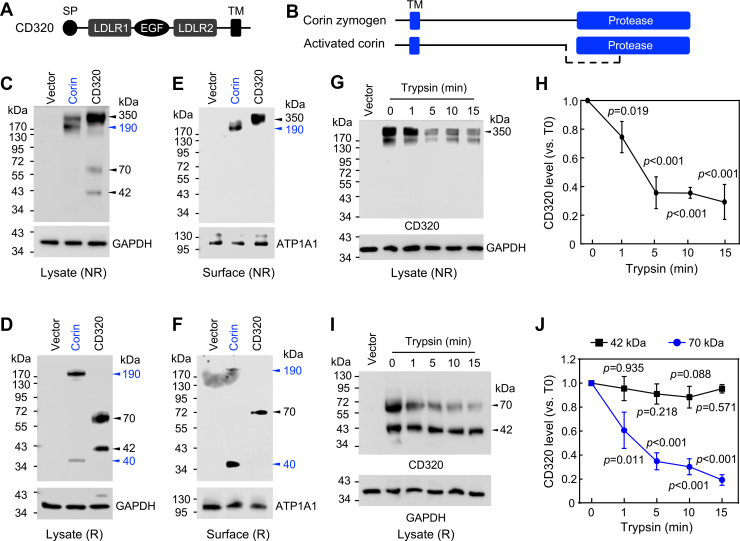
**Expression of human CD320 oligomers in transfected HEK293 cells.***A*, schematic presentation of CD320 protein domains. SP, signal peptide; TM, transmembrane domain. *B*, illustration of corin protein before and after zymogen activation. The protease domain is linked to the propeptide by a disulfide bond (dashed line) after activation cleavage. *C* and *D*, Western blotting of lysates from HEK293 cells transfected with a vector or plasmids expressing V5-tagged human corin (control) and FLAG-tagged CD320 under non-reducing (NR) (*C*) and reducing (R) (*D*) conditions using anti-V5 and anti-FLAG antibodies. GAPDH was a sample loading control. *E* and *F*, Western blotting of CD320 and corin in biotinylated cell surface proteins under NR (*D*) and R (*E*) conditions. Na^+^/K^+^ ATPase 1 (ATP1A1) was a membrane protein control. *G*–*J*, HEK293 cells expressing CD320 were treated with trypsin over time and lysed for western blotting under NR (*G* and *H*) and R (*I* and *J*) conditions. Levels of CD320 bands on western blots were quantified by densitometry. The data are representative of at least three experiments. *p* values vs. 0 min in trypsin digestion analyzed by one-way ANOVA are indicated.

CD320 consists of an N-terminal signal peptide, an extracellular region with two LDLR-like domains and an EGF-like domain, a TM domain, and a C-terminal cytoplasmic tail (Fig. 1*A*). We analyzed human CD320 protein forms in HEK293 cells. In these experiments, we included corin, a membrane-bound protease that undergoes activation cleavage on the cell surface (32, 33). The C-terminal protease domain of the activated corin is tethered to the propeptide by a disulfide bond (Fig. 1*B*), which can be used as an indicator for corin cell surface localization and activation (33, 34).

We transfected HEK293 cells with plasmids expressing corin and CD320 or a control vector and analyzed cell lysates by western blotting. Under non-reducing conditions, we detected corin bands of ∼190 to 200 kDa and CD320 bands of ∼42, ∼70, and ∼350 kDa, respectively (Fig. 1*C*). In the vector transfected cells, no bands were detected (Fig. 1*C*). Under reducing conditions, we detected ∼40- and ∼190-kDa corin bands, representing the cleaved protease domain and the corin zymogen, respectively (Fig. 1*D*). CD320 was detected only as ∼42- and ∼70-kDa bands (Fig. 1*D*). The results were consistent with our previous findings (31), indicating that β-mercaptoethanol dissociated the ∼350-kDa CD320 oligomer, giving two low-molecular units. The smaller ∼42-kDa unit represented the N-glycosylated CD320 monomer (processed in the endoplasmic reticulum, ER), while the larger ∼70-kDa unit represented the N- and O-glycosylated CD320 monomer (additionally processed in the Golgi).

We next examined CD320 on the cell surface. Cell surface proteins were biotin-labeled and analyzed by western blotting. Under non-reducing and reducing conditions, CD320 was detected as a ∼350-kDa (Fig. 1*E*) and a ∼70-kDa (Fig. 1*F*) band, respectively. These results indicated that CD320 was present on the cell surface in an oligomeric form, which consisted of the ∼70-kDa monomers. As expected, corin was detected as a ∼190-kDa band under non-reducing conditions (Fig. 1*E*) and ∼40- and ∼190-kDa bands under reducing conditions (Fig. 1*F*). Afterward, we treated the transfected cells with trypsin to remove cell surface proteins and then lysed the cells for western blotting. As shown in western blotting under non-reducing conditions, levels of the ∼350-kDa CD320 band were reduced to ∼25 to 30% after 15 min of trypsin treatment (Fig. 1, *G* and *H*). In western blotting under reducing conditions, levels of the ∼70-kDa CD320 band, which was expected to be in the Golgi and on the cell surface, were reduced to ∼20% after 15 min of trypsin treatment, whereas levels of the ∼42-kDa CD320 band, which was expected to be in the ER, were not reduced significantly (Fig. 1, *I* and *J*). These results indicated that CD320 oligomerization occurred intracellularly before reaching the cell surface.

### CD320 oligomers are formed in the ER

**Figure 2:**
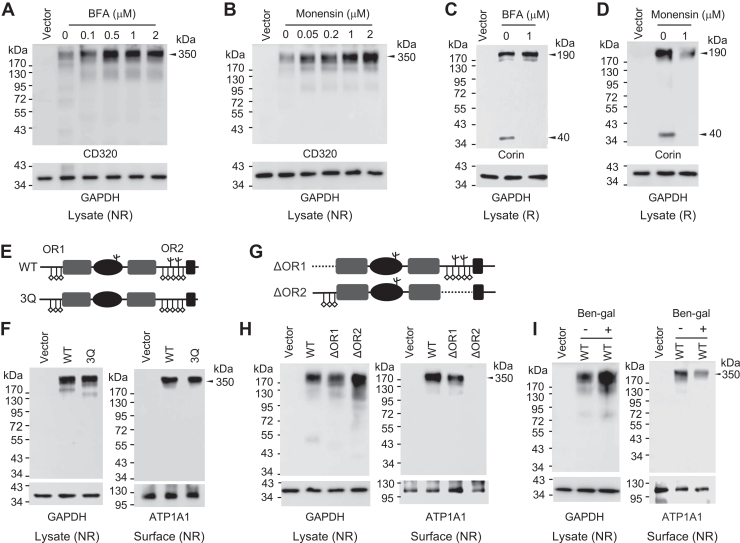
**Effects of BFA, monensin and glycosylation on CD320 oligomer formation.***A* and *B*, HEK293 cells expressing CD320 were treated with BFA (*A*) or monensin (*B*) at indicated concentrations. Western blotting of CD320 in cell lysates was done under non-reducing (NR) conditions. GAPDH was a sample loading control. *C* and *D*, as controls, HEK293 cells expressing corin were treated with BFA (*C*) or monensin (*D*). Western blotting of corin in cell lysates was done under reducing (R) conditions. The ∼40-kDa band of the protease domain was an indicator of activated corin on the cell surface. *E*, Illustration of N-glycosylation sites (y shaped symbols) and O-glycan-rich (OR) regions (diamonds) in CD320 WT and the 3Q mutant. *F*, Western blotting of CD320 in lysates (*left*) and surface proteins (*right*) from transfected HEK293 cells. *G*, illustration of the CD320 mutants lacking the OR1 (ΔOR1) or OR2 (ΔOR2) regions. *H*, Western blotting of CD320 WT and the mutants in lysates (*left*) and surface proteins (*right*) from the transfected cells. *I*, Western blotting of CD320 WT in lysates (*left*) and surface proteins (*right*) from the transfected cells in the absence (−) or presence (+) of Ben-gal. The data are representative of at least three experiments.

To understand the subcellular location where CD320 oligomerization occurred, we treated the transfected cells with brefeldin A (BFA), which blocks protein trafficking from the ER to the Golgi, and monensin, which blocks protein trafficking from the Golgi to the cell surface. In western blotting of cell lysates under non-reducing conditions, levels of the ∼350-kDa CD320 band appeared increased after BFA or monensin treatment (Fig. 2, *A* and *B*). In controls, the ∼40-kDa corin protease domain band was not detected in the BFA- or monensin-treated cells (Fig. 2, *C* and *D*), consistent with previous reports that BFA and monensin blocked intracellular protein trafficking, preventing corin activation on the cell surface (33). These results indicated that blocking protein intracellular trafficking caused accumulation of CD320 oligomers in the ER and the Golgi.

### N- and O-glycans are not required for CD320 oligomer formation

Glycans play an important role in protein folding, trafficking, and oligomerization (35, 36, 37). Human CD320 is N-glycosylated at Asn126 in the EGF domain and Asn195 and Asn213 in the second O-glycan-rich (OR2) region (Fig. 2*E*). Substitution of Asn with Gln residues at these sites abolishes N-glycosylation in CD320 (31). To test if N-glycans are required for CD320 oligomerization, we analyzed the CD320 mutant 3Q, in which Asn126, Asn195 and Asn213 were replaced with Gln residues (Fig. 2*E*). In western blotting under non-reducing conditions, similar levels of the ∼350-kDa band were detected in lysates and surface proteins from the transfected cells expressing CD320 wild-type (WT) and the mutant 3Q (Fig. 2*F*).

O-glycosylation is crucial for CD320 localization on the cell surface (31). O-glycans in human CD320 are clustered primarily in two regions: O-glycan-rich 1 (OR1) near the N-terminus and OR2 between the LDLR2 and TM domains (Fig. 2*E*). To test the importance of O-glycans in CD320 oligomerization, we analyzed the CD320 mutants ΔOR1 and ΔOR2, in which the OR1 and OR2 regions were deleted, separately (Fig. 2*G*). In western blotting under non-reducing conditions, the ∼350-kDa band was detected in lysates from the cells expressing CD320 WT and the mutants ΔOR1 and ΔOR2 (Fig. 2*H*, *left panel*). Levels of the ∼350-kDa band were similar in WT and the mutant ΔOR1, but higher in the mutant ΔOR2. The ∼350-kDa band was also detected in biotin-labeled surface proteins from the cells expressing CD320 WT and the mutant ΔOR1, but not the mutant ΔOR2 (Fig. 2*H*, *right panel*).

As reported (31), O-glycans in the OR2 region are required for CD320 trafficking in the Golgi network. The increased levels of the ∼350-kDa band in lysates and the absence of the band on the cell surface in the cells expressing the mutant ΔOR2 indicated that lacking O-glycosylation in the OR2 region caused intracellular retention of CD320 oligomers. To verify this result, we treated the cells expressing WT CD320 with benzyl N-acetyl-α-D-galactosaminide (Ben-gal), an analog of GalNAc-α-1-O-Ser/Thr that prevents O-glycan elongation (38, 39). As shown in western blotting under non-reduction conditions, Ben-gal treatment increased levels of the ∼350-kDa CD320 band in lysates (Fig. 2*I*, *left panel*), but decreased levels of the CD320 band in surface proteins (Fig. 2*I, right panel*), indicating that the Ben-gal treatment blocked CD320 intracellular trafficking but not oligomerization.

### The CD320 oligomer is shed from the cell surface

**Figure 3:**
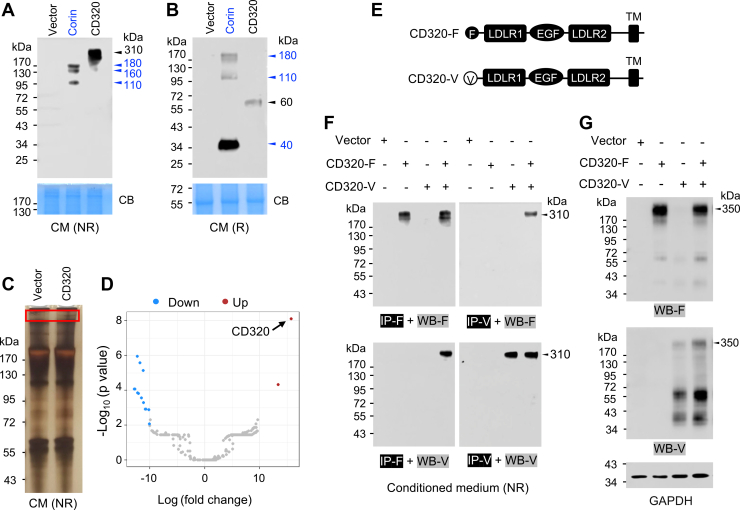
**Analysis of CD320 oligomers in the conditioned media.***A* and *B*, immunoprecipitation and western blotting of V5-tagged corin (control) and CD320 in the conditioned media from transfected HEK293 cells under non-reducing (NR) (*A*) and reducing (R) (*B*) conditions. Coomassie *blue* (CB)-stained non-specific bands in the gel were used as sample loading controls. *C*, image of the silver-stained gel, from which a section (*red* box) was used for proteomic analysis with LC-MS. *D*, Volcano plot showing CD320 as the most enriched protein in the conditioned media from CD320-expressing vs. vector-transfected cells. *E*, illustration of FLAG (F) or V5 (V)-tagged CD320. *F* and *G*, FLAG- and V5-tagged CD320 proteins were expressed in HEK293 cells, alone or together. Immunoprecipitation (IF) and western blotting (WB) were done using anti-FLAG and V5 antibodies to examine CD320 proteins in the conditioned media (*F*) and cell lysates (*G*). The data of IP and WB were representative of at least three experiments.

Ectodomain shedding is common among cell surface proteins (40, 41, 42). To examine if the CD320 oligomer undergoes ectodomain shedding, we analyzed the conditioned media from the cells expressing WT CD320 or corin (control). In western blotting, a ∼310-kDa band and a ∼60-kDa band of CD320 were detected under non-reducing and reducing conditions, respectively (Fig. 3, *A* and *B*), thereby testifying for CD320 shedding. As reported previously (34, 43), corin fragments cleaved by metalloproteinases and autocatalysis were detected under non-reducing and reducing conditions (Fig. 3, *A* and *B*).

### The CD320 oligomer is made of homomers

To understand the oligomeric nature of CD320, we collected the conditioned media from the cells expressing WT CD320 or vector-transfected cells, separated proteins with SDS-PAGE, and silver-stained the gel. A section at the ∼310-350-kDa position in the gel was cut (Fig. 3*C*) and subjected to proteomic analysis using liquid chromatography-mass spectrometry (LC-MS). The results showed that CD320 was the only protein detected, which was highly enriched in the conditioned medium from the CD320 expressing cells, compared to the sample from the vector transfected cells (Fig. 3*D*). These results indicated that the CD320 oligomer consisted of homomers, although the possibility could not be excluded that small protein(s) might be dissociated from the CD320 oligomer in the presence of SDS.

To verify the conjecture about the homomeric nature of CD320 oligomers, we expressed CD320 with an N-terminal FLAG (CD320-F) or V5 tag (CD320-V) (Fig. 3*E*) in HEK293 cells. The conditioned media were collected. Immunoprecipitation was carried out using either an anti-FLAG antibody (IP-F) (Fig. 3*F*, *left top and bottom panels*) or an anti-V5 antibody (IP-V) (Fig. 3*F*, *right top and bottom panels*). The precipitated proteins were examined by western blotting using either an anti-FLAG antibody (WB-F) (Fig. 3*F*, *top left and right panels*) or an anti-V5 antibody (WB-V) (Fig. 3*F*, *bottom left and right panels*).

As shown in the top left panel of Figure 3*F*, CD320-F was pulled down from the conditioned medium and detected on western blot (second and fourth lanes) by the anti-FLAG antibody (IP-F + WB-F), as expected. The top right panel of Figure 3*F* showed that the anti-V5 antibody pulled down CD320-V in the conditioned medium, and the anti-FLAG antibody detected CD320-F (fourth lane) on the western blot (IP-V + WB-F), indicating that the original monomers of CD320-V and CD320-F were present as a mixed oligomer in the conditioned medium. Likewise, when immunoprecipitation was done using the anti-FLAG antibody followed by western blotting with the anti-V5 antibody (IP-F + WB-V) (Fig. 3*F*, *bottom left panel*), CD320-V was detected (fourth lane), another indication of the CD320-V/CD320-F complex. When immunoprecipitation and western blotting were done using the anti-V5 antibody (IP-V + WB-V) (Fig. 3*F*, *bottom right panel*), CD320-V was detected on western blot (third and fourth lanes), as expected. As controls, CD320-F and CD320-V proteins were confirmed in lysates from the transfected cells (Fig. 3*G*). These results indicated that overexpression of CD320 in the transfected cells was sufficient to produce interreacted CD320 homo-oligomers and that, apparently, no other proteins were required for the oligomer formation.

### A potential CD320 pentamer structure is predicted by AlphaFold3

Given the homomeric nature, the CD320 oligomer is likely a pentamer, considering the observed molecular masses of the oligomer in lysates (∼350 kDa) and the conditioned media (∼310 kDa), which were approximately five times of the monomers in lysates (∼70 kDa) and the conditioned media (∼60 kDa), respectively. We used AlphaFold3 software to predict potential three-dimensional (3D) pentamer structure of CD320 (44). Based on the amino acid sequence of the human CD320 extracellular region, five CD320 monomers were predicted to form a symmetrical cage-like structure with a pore at the center, as viewed from the top (Fig. S1*A*). The LDLR domains formed the interface between the monomers with the LDLR1 domain of one monomer interacting with the LDLR2 domain of another monomer. The EGF domain between the LDLR domains were protruding outward in the peripheral. From the side view, the OR1, LDLR1/2, and EGF domains occupied the top part of the cage-like structure, whereas the OR2 regions formed the bottom part of the structure (Fig. S1*B*). In this exploratory 3D model, potential intermolecular disulfide bonds, which were indicated in the western blotting experiments, were not predicted.

### LDLR1 and LDLR2 domains are crucial for CD320 oligomerization

**Figure 4:**
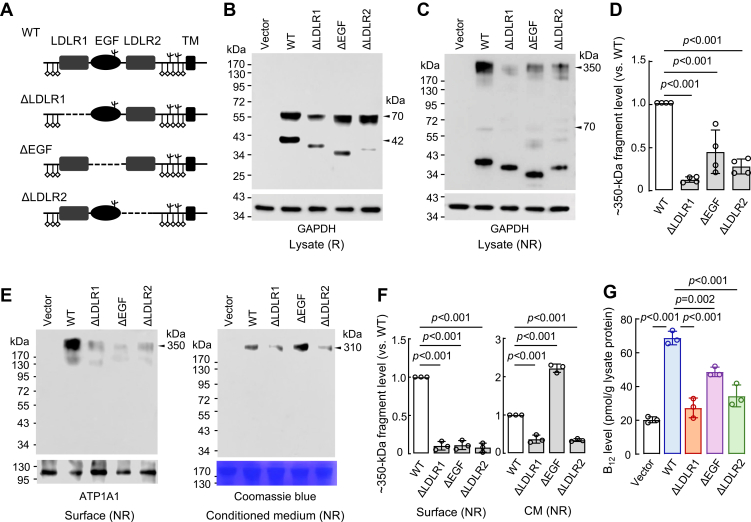
**Importance of CD320 domains in oligomer formation and vitamin B_12_ uptake.***A*, illustration of the CD320 mutants in which the LDLR1, EGF and LDLR2 domains were deleted individually. *B* and *C*, Western blotting of CD320 WT and the deletion mutants in HEK293 cell lysates under reducing (R) (*B*) and non-reducing (NR) (*C*) conditions. *D*, quantification of the ∼350-kDa band on western blots under NR conditions (n = 4). *E* and *F*, Western blotting of CD320 WT and the deletion mutants in cell surface proteins (*left*) and the conditioned media (*right*) under NR conditions (*E*). Levels of the ∼350-kDa band on western blots were quantified by densitometry (*F*). *G*, Vitamin B_12_ levels in lysates from transfected HepG2 cells expressing CD320 WT and the deletion mutants. The data in *D*, *F* and *G* are mean ± S.D. analyzed by one-way ANOVA (n = 3). The data in (*D*) and (*F*) were independent biological replicates and the data in (*G*) were technical replicates.

As shown in Figure 2*H*, the OR1 and OR2 regions were not required for CD320 oligomerization. We tested the importance of the LDLR1, EGF, and LDLR2 domains in CD320 oligomerization. We made the CD320 mutants ΔLDLR1, ΔEGF and ΔLDLR2, in which the LDLR1, EGF and LDLR2 domains were deleted individually (Fig. 4*A*). The mutants were expressed in HEK293 cells and analyzed by western blotting under reducing conditions. The N-glycosylated (∼36–42 kDa) and N- and O-glycosylated (∼60–70 kDa) CD320 bands were detected in lysates from the cells expressing CD320 WT and the mutants (Fig. 4*B*). In western blotting under non-reducing conditions, levels of the ∼350-kDa band were much lower in the ΔLDLR1, ΔEGF and ΔLDLR2 mutants than those of WT (Fig. 4, *C* and *D*). Similarly, reduced levels of the ∼350-kDa band were observed in surface proteins from the cells expressing the deletion mutants (Fig. 4, *E* and *F, left panels*). We also examined CD320 fragments in the conditioned media by western blotting. Reduced levels of the ∼310-kDa band were detected in samples from the ΔLDLR1 and ΔLDLR2 mutants, whereas increased levels of the ∼310-kDa band were observed in samples from the ΔEGF mutant (Fig. 4, *E* and *F*, *right panels*). These results indicated that the deletion of the LDLR1 or LDLR2 domain inhibited CD320 oligomerization, whereas the deletion of the EGF domain did not prevent the oligomerization but enhanced CD320 shedding from the cell surface. In addition, we also found reduced vitamin B_12_ uptake in HepG2 cells expressing the mutants ΔLDLR1, ΔEGF and ΔLDLR2, compared to that in the cells expressing CD320 WT (Fig. 4*G*).

**Figure 5:**
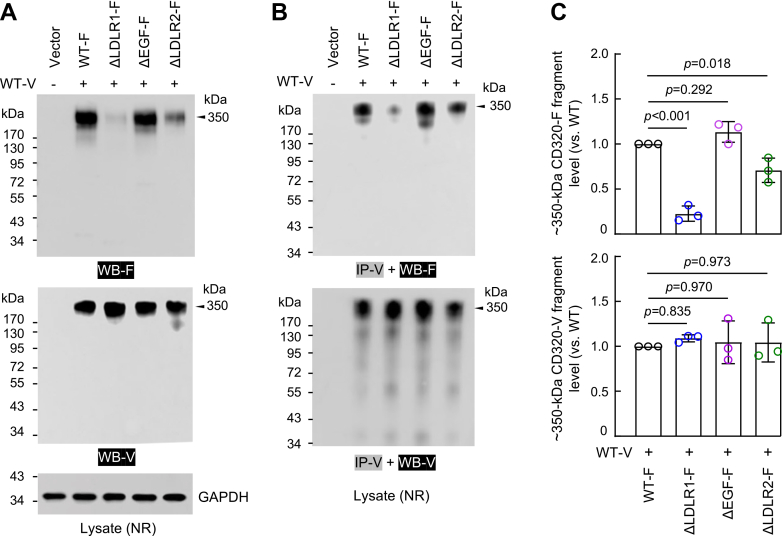
**Intermolecular interactions between CD320 monomers.***A*, HEK293 cells were transfected with plasmids expressing V5-tagged CD320 WT (WT-V) and FLAG-tagged CD320 WT (WT-F) or the deletion mutants ΔLDLR1 (ΔLDLR1-F), ΔEGF (ΔEGF-F) or ΔLDLR2 (ΔLDLR2-F). Cell lysates were prepared and western blotting was done under non-reducing (NR) conditions using an anti-FLAG (WB-F) (*top*) or anti-V5 (WB-V) (*middle*) antibody. GAPDH was a sample loading control (*bottom*). *B*, V5-tagged CD320 WT was immunoprecipitated using an anti-V5 antibody (IP-V). Proteins pulled down by the anti-V5 antibody were examined by western blotting using an anti-FLAG (WB-F) (*top*) or anti-V5 (WB-V) (*bottom*) antibody. *C*, Levels of the ∼350-kDa CD320-F (*top*) or CD320-V (*bottom*) bands on western blots in (*B*) were quantified by densitometry. The data are mean ± S.D. analyzed by one-way ANOVA (n = 3). The data in (*C*) were independent biological replicates.

To test potential interactions among the LDLR1, EGF and LDLR2 domains, we co-expressed V5-tagged CD320 WT and FLAG-tagged CD320 WT and the mutants ΔLDLR1, ΔEGF and ΔLDLR2 in HEK293 cells. Western blotting of FLAG-tagged CD320 showed reduced levels of the ∼350-kDa band in the mutants ΔLDLR1-F and ΔLDLR2-F, but not the mutant ΔEGF-F, when compared to WT-F (Fig. 5*A*, *top panel*). As controls, levels of the ∼350-kDa band of CD320 WT-V (Fig. 5*A*, *middle panel*) and GAPDH (Fig. 5*A*, *bottom panel*) were comparable among the samples. The results indicated that CD320 oligomerization was impaired in the mutants ΔLDLR1-F and ΔLDLR2-F, but not in ΔEGF-F, suggesting that in the presence of CD320 WT-V, ΔEGF-F oligomers were stabilized.

We next did immunoprecipitation using an anti-V5 antibody, followed by western blotting with an anti-FLAG antibody. Reduced levels of the ∼350-kDa band were found in the mutants ΔLDLR1 and ΔLDLR2 but not the mutant ΔEGF (Fig. 5, *B* and *C*, *top panels*). When western blotting was done with an anti-V5 antibody (IP-V + WB-V), similar levels of the ∼350-kDa band were found in all samples pulled down by the anti-V5 antibody (Fig. 5, *B* and *C*, *bottom panels*). These results indicated that intermolecular interactions among LDLR1/2 domains in CD320-V and CD320-F may occur during CD320 oligomerization.

### The TM domain is required for CD320 oligomerization

**Figure 6:**
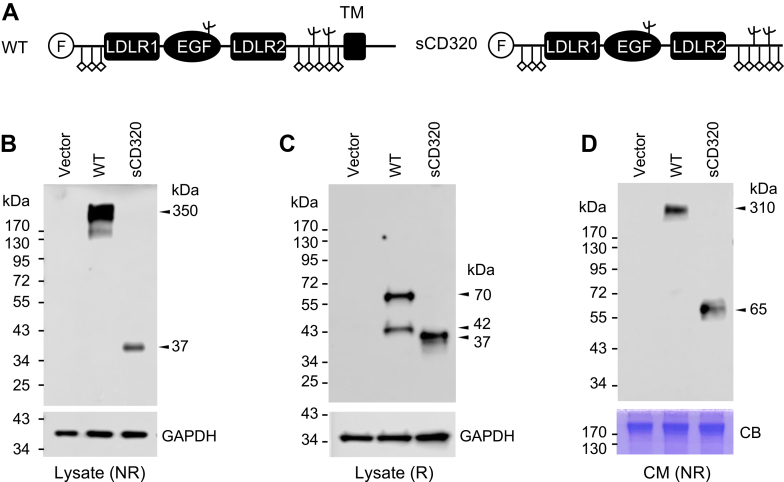
**Analysis of sCD320 in cell lysates and the conditioned medium.***A*, illustration of FLAG (F)-tagged CD320 WT and sCD320 consisting of the full-length extracellular region. *B*–*D*, CD320 WT and sCD320 were expressed in HEK293 cells. Western blotting of CD320 proteins in cell lysates was done using an anti-FLAG antibody under non-reducing (NR) (*B*) and reducing (R) (*C*) conditions. GAPDH was a sample loading control. CD320 proteins in the conditioned media from the cells expressing CD320 WT and sCD320 were analyzed by western blotting using an anti-FLAG antibody under NR conditions. A Coomassie blue (CB) stained non-specific band was used as a sample control (*D*). The data are representative of three experiments.

To test the importance of the TM domain in CD320 oligomerization, we made a plasmid expressing a soluble form of CD320 (sCD320) that included the full-length extracellular region without the TM domain and the cytoplasmic tail (Fig. 6*A*). CD320 WT and sCD320 were expressed in HEK293 cells and analyzed by western blotting. The ∼350-kDa band was detected under non-reducing conditions, and the ∼70- and ∼42-kDa bands were detected under reducing conditions in CD320 WT (Fig. 6, *B* and *C*). In contrast, only a ∼37-kDa band of sCD320 was detected under non-reducing and reducing conditions in cell lysates, which likely represented intracellular sCD320 monomer (Fig. 6, *B* and *C*). In the conditioned medium from the cells expressing sCD320, only a ∼65-kDa band, likely representing glycosylated sCD320 monomer, was detected in western blotting under non-reducing conditions.

### The ΔE88 variant impairs CD320 intracellular trafficking and cellular uptake of vitamin B_12_

**Figure 7:**
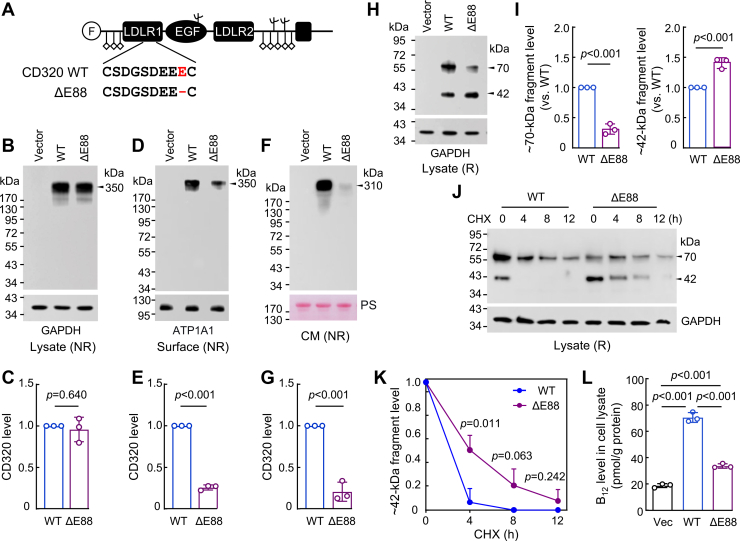
**Effects of the ΔE88 variant on CD320 oligomer formation and intracellular trafficking.***A*, illustration of CD320 WT and the ΔE88 variant. *B*–*G*, Western blotting of CD320 proteins with a FLAG (F) in lysates (*B* and *C*), surface proteins (*D* and *E*), and the conditioned media (*F* and *G*) under non-reducing (NR) conditions. GAPDH, ATP1A1 and Ponceau S (PS)-stained non-specific bands were controls. Levels of the CD320 bands on western blots were quantified. The data of mean ± S.D. in (*C*), (*E*), and (*G*) were analyzed by Student’s *t* test (n = 3). *H* and *I*, Western blotting of CD320 proteins in cell lysates under reducing (R) conditions. Levels of the ∼70- and ∼42-kDa bands on western blots were quantified. *J* and *K*, HEK293 cells expressing CD320 proteins were treated with cycloheximide (CHX) and lysed at different times. Western blotting of CD320 proteins was done using an anti-FLAG antibody under reducing (R) conditions. Levels of the ∼42-kDa band were quantified by densitometry and plotted in (K). *p* values vs. WT at the same time point after CHX treatment were analyzed by Student’s *t* test. *L*, Vitamin B_12_ levels were measured by ELISA in lysates from HepG2 cells transfected with a control vector or a plasmid expressing CD320 WT or the ΔE88 variant. The data are mean ± SD analyzed by one-way ANOVA (n = 3). The data in (*C*), (*E*), (*G*) and (*I*) were independent biological replicates, and the data in (*L*) were technical replicates.

The *CD320* variant NM_016579.3:c.262_264delGAG is a naturally occurring allele with an in-frame deletion of three base pairs encoding a Glu residue near the C-terminus of the LDLR1 domain, referring to as ΔE88 hereafter (Fig. 7*A*). Infants with this variant exhibit methylmalonic acidemia and increased circulating vitamin B_12_ levels (45, 46, 47). To date, the cellular mechanism underlying the apparent dysfunction of the ΔE88 variant remained unclear. To test if the ΔE88 variant impairs CD320 oligomerization, we expressed CD320 WT and the ΔE88 variant in HEK293 cells. Western blotting under non-reducing conditions showed comparable levels of the ∼350-kDa band in lysates from the cells expressing CD320 WT and the ΔE88 variant (Fig. 7, *B* and *C*). However, levels of the ∼350-kDa band in surface-labeled proteins (Fig. 7, *D* and *E*) and the ∼310-kDa band in the conditioned medium (Fig. 7, *F* and *G*) were markedly decreased in the cells expressing the ΔE88 variant, pointing to impaired trafficking of this mutant to the cell surface.

Consistent with these findings, western blotting under reducing conditions showed that the ΔE88 variant had reduced levels of the ∼70-kDa band, representing the N- and O-glycosylated CD320 in the Golgi or cell surface (31), but increased levels of the ∼42-kDa band, representing the N-glycosylated CD320 in the ER (31) (Fig. 7, *H* and *I*). We next did a cycloheximide (CHX) chase experiment to study CD320 intracellular trafficking. As shown in western blotting under reducing conditions, the ∼42-kDa band from WT CD320 disappeared < 4 h after the CHX treatment, whereas this band from the ΔE88 variant remained detectable for up to 12 h after the CHX treatment (Fig. 7, *J* and *K*). These results indicated that the ΔE88 variant had delayed trafficking from the ER to the cell surface. In line with these results, reduced vitamin B_12_ uptake was observed in the cells expressing the ΔE88 variant, compared to that in the CD320 WT expressing cells (Fig. 7*L*), indicating that poor intracellular trafficking and low cell surface localization of the ΔE88 variant impaired cellular uptake of vitamin B_12_.

## Discussion

CD320 is the primary receptor for the cellular uptake of circulating holo-TC. In early biochemical studies with solubilized cell membrane fractions from human placentae, CD320 was found to be a monomeric receptor (29, 30). In this study, we show that CD320 more likely exists as an oligomeric receptor on the cell surface. This conclusion is based on several lines of experimental evidence. In western blotting under non-reducing conditions, only a ∼350-kDa band of oligomeric CD320 was detected on the cell surface, whereas under reducing conditions, only a ∼70-kDa band of monomeric CD320 was detected, indicating that CD320 was present on the cell surface in an oligomeric form that consisted of monomers. In line with these results, only a ∼310-kDa band of oligomeric CD320, but no monomeric CD320, was found in western blotting under non-reducing conditions in the conditioned medium from CD320-expressing cells. These results indicate that CD320 is present as oligomers but not monomers on the cell surface. In line with our findings, a high-molecular (above the 220-kDa marker) CD320 band was detected but not further characterized in human placental membrane fractions (48). Our findings also suggest that ectodomain shedding may be a potential mechanism in regulating CD320 function, consistent with previous reports of soluble CD320 in human blood, urine and cerebrospinal fluid under pathophysiological conditions (49, 50, 51, 52).

In a previous study, crystal structures of human CD320-TC complex were determined, in which no CD320 oligomers were reported (15). The experiments were done using extracellular fragments of human CD320 (residues 31–199 and 53–199, respectively) without the TM domain. In our study, we found that CD320 oligomerization occurred in the ER, as BFA and monensin treatment, which blocked protein trafficking in the ER and the Golgi, respectively, did not prevent the CD320 oligomerization. Importantly, we showed that the TM domain was necessary for CD320 oligomerization, as indicated by the absence of CD320 oligomers, either in cell lysates or the conditioned medium, when sCD320 was expressed in the transfected cells. In principle, the deletion of the TM domain may alter CD320 topology on the ER membrane, making sCD320 a cytosolic protein and hence preventing its oligomerization in the ER. Should this be the case, sCD320 is expected to remain in the cytosol. In our study, sCD320 was detected as a ∼65-kDa monomer in the conditioned medium under non-reducing conditions, indicating that sCD320 was a secreted but not cytosolic protein. Together, these results could explain why no CD320 oligomers were observed in the crystal structures of the TM domain-less human CD320 extracellular fragments complexed with TC (15).

Oligomerization is a common process among cell membrane receptors, contributing to ligand binding, signal transduction, and endocytosis (53, 54). In our western blotting analyses, the observed molecular masses of the CD320 oligomer (∼350 kDa) were approximately five times greater than those of the CD320 monomer (∼70 kDa), indicating that the CD320 oligomer likely consisted of five homomers. The immunoprecipitation experiments with FLAG- and V5-tagged CD320 proteins also indicated that the CD320 oligomer was homomeric, but not heteromeric. In the western blotting experiments, the ∼350-kDa CD320 oligomer was not observed under reducing conditions that disrupted disulfide bonds, indicating that intermolecular disulfide bonds were involved in the formation of CD320 oligomers. The results from the LC-MS analysis also supported the idea that CD320 oligomers consisted of homomers. However, the LC-MS experiment was done with gel samples from SDS-PAGE, in which reversible protein-protein interactions are expected to be disrupted. Thus, potential reversible association of other small protein(s) with the CD320 oligomers could not be ruled out.

In the exploratory 3D model of the CD320 oligomer predicted by AlphaFold3, five CD320 homomers formed a symmetrical cage-like structure with a central pore that could open upon ligand binding. Interactions between neighboring homomers appeared to be mediated primarily *via* the LDLR domains. This 3D model, however, did not provide information regarding potential intermolecular disulfide bonds that were indicated by the western blotting experiments. In transfected cells, the CD320 mutants lacking either the LDLR1 or LDLR2 domain failed to form oligomers. The immunoprecipitation and western blotting experiments with V5- and FLAG-tagged CD320 WT and the ΔLDLR1 and ΔLDLR2 mutants indicated possible interactions among the LDLR domains in separate CD320 homomers. In principle, the EGF domain and the OR2 segment may contribute to the formation and/or stability of the pentamer. In our study, however, the ΔEGF mutant exhibited increased shedding from the cell surface, but not diminished oligomerization, as indicated by higher levels of the oligomerized ∼310-kDa fragment in the conditioned medium. The ΔOR2 mutant also formed oligomers intracellularly, although its trafficking to the cell surface was blocked, indicating that O-glycans are critical for CD320 intracellular trafficking but dispensable for CD320 oligomerization in the ER. Currently, the AlphaFold3 model of CD320 oligomers remains exploratory, and its biological relevance is uncertain. In the published human CD320 crystal structure (15), the LDLR1 and LDLR2 domains of CD320 interact with holo-TC, which is expected to hinder potential interactions among CD320 monomers. Further structural biology studies are required to verify if CD320 indeed exists as pentamers on the cell surface and how individual CD320 domains participate in the oligomer assembly.

The CD320 ΔE88 variant was identified in infants with methylmalonic aciduria, a metabolic disorder associated with abnormal vitamin B_12_ function (45, 46, 47). *In vitro* studies with fibroblasts from affected individuals showed a reduction in CD320-mediated binding and uptake of holo-TC (47). Consistently, the fibroblasts from these individuals exhibited low activities of cytoplasmic methionine synthase and mitochondrial methylmalonyl-CoA mutase, which are vitamin B_12_-dependent (47). Curiously, recombinant CD320 ΔE88 variant had similar thermal stability and binding affinity for TC, compared with WT CD320, when tested *in vitro* (15). In polarized Madin-Darby canine kidney cells, the expression of the ΔE88 variant was detected on the apical membrane; however, the finding was qualitative but not quantitative (47). Possibly, the ΔE88 variant may impair other cellular mechanisms that regulate CD320 biosynthesis and/or function. In this study, we showed that CD320 oligomerization was not impaired in the ΔE88 variant. Instead, the variant displayed poor trafficking in the ER, resulting in low levels on the cell surface. These results help reconcile findings from the previous studies, providing new insights into the cellular mechanism underlying the defects of the ΔE88 variant in vitamin B_12_ metabolism.

Our results from the CD320 ΔE88 variant share intriguing similarities with findings from other cell surface proteins with LDLR-like repeats. For example, the mutation NM_000527.4: c.654_656delTGG or p.Gly219del is a 3-base-pair in-frame deletion in exon four of the *LDLR* gene, resulting in a mutant LDLR protein lacking Gly219 in an LDLR class-A repeat (55, 56, 57). The mutation impairs protein folding and delays ER trafficking to the Golgi, resulting in low LDLR expression on the cell surface. Individuals with this mutation have < 5% of normal LDLR activity, causing familial hypercholesterolemia (55, 56, 57). In the human *CORIN* gene, encoding the transmembrane protease corin essential for salt-water balance and normal blood pressure, mutations altering amino acids in the LDLR-like domains impair protein conformation and function, contributing to hypertension and heart disease (58, 59, 60, 61). These results highlight the importance of the LDLR-like domains in human transmembrane proteins that are of major physiological importance.

In summary, CD320 plays a pivotal role in vitamin B_12_ metabolism. In this study, we show that CD320 is present as homo-oligomers on the cell surface. In transfected cells, impaired oligomerization reduces CD320 levels on the cell surface and cellular uptake of vitamin B_12_. Our findings challenge the prevailing view of CD320 as a monomeric receptor. By analyzing CD320 forms in intracellular compartments, we show that CD320 oligomerization occurs in the ER and that the process requires the TM domain, but not N- or O-glycans, of CD320. Moreover, we show that the ΔE88 variant does not prevent CD320 oligomerization but impairs protein trafficking from the ER to the cell surface. Together, these findings offer important insights into the biochemical nature and cellular mechanisms underlying the function of CD320 and associated pathologies in vitamin B_12_ metabolism.

## Experimental procedures

### Expression plasmids

The plasmids expressing human CD320 WT (NCBI accession number: NP_057663.1), the mutants 3Q (N126Q/N195Q/N213Q), ΔOR1 (deleting residues 36–52), and ΔOR2 (deleting residues 170–230), and the ΔE88 variant were reported previously (19, 31, 47). Additional CD320 mutants, including ΔLDLR1 (deleting residues 53–90), ΔEGF (deleting residues 91–130), ΔLDLR2 (deleting residues 131–168), and sCD320 (consisting of the full-length extracellular region, residues 36–230), were made by site-directed mutagenesis using ClonExpress One Step Cloning Kit (Vazyme, C112). The recombinant CD320 included a FLAG or V5 tag at the N-terminus. The plasmid expressing human corin WT with a C-terminal V5 tag was reported previously (34). All plasmids used in this study were confirmed by DNA sequencing.

### Cell culture and plasmid transfection

HEK293 (CRL-1573) and HepG2 (HB-8065) cells from American Type Culture Collection (authenticated by short tandem repeat profiling) were cultured in Dulbecco’s modified Eagle’s medium (DMEM) (Corning, 10–0130CVRC) with 10% fetal bovine serum (FBS) (Sigma, F0193) at 37 °C with 5% CO_2_. When the cells reached at ∼80% confluency, plasmids were transfected into the cells using PolyJet In Vitro DNA Transfection Reagent (SigmaGen Laboratories, SL100688). After 6 h at 37 °C, fresh medium was added and the cells were incubated for additional 16 to 48 h before being collected for further biochemical analyses.

### Western blotting

To analyzed CD320 proteins, the transfected HEK293 cells were washed with phosphate buffered saline (PBS) and lysed in a Tris-HCI solution (pH 8.0) with 1% Triton X-100, 150 mM NaCl and protease inhibitors (1:100, Roche Applied Science, 04693116001). Protein levels in the lysates were measured by a bicinchoninic acid assay (P0012, Beyotime). For western blotting, the protein samples were mixed in Laemmli buffer (Bio-Rad, 161–0737) without (non-reducing) or with (reducing) 2.5% (v/v) β-mercaptoethanol, run with SDS-PAGE, and transferred to polyvinylidene difluoride membranes. The blots were incubated with horseradish peroxidase (HRP)-conjugated antibodies against FLAG (1:10,000, Sigma, A8592) or V5 (1:5000, Thermo Fisher, R96125) tags. To assess proper protein loading, the blots were re-probed with an antibody against glyceraldehyde-3-phosphate dehydrogenase (GAPDH) (1:10,000, Boster Bio, A00227–1) for proteins in cell lysates or an antibody against Na+/K + ATPase 1 (ATP1A1) (1:1000, ZenBio, R380790) for cell surface proteins, followed by an HRP-conjugated secondary antibody (1:10,000, Boster Bio, BA1054). After washing, the blots were treated with a chemiluminescent agent (NCM Biotech, P10050) and analyzed with Amersham Imager 600. Protein bands were quantified by densitometry. For proteins in the conditioned media, Coomassie Blue (CB) and Ponceau S (PS)-stained non-specific protein bands in the get and blots were used, respectively, to indicate protein sample loading.

### Examination of cell surface proteins

To analyze proteins on the cell surface, the transfected HEK293 cells were incubated with Sulfo-NHS-SS-biotin (0.25 mg/ml) (Thermo Fisher, 89,881) on ice for 4 min. A glycine solution (100 mM) was added to the cells to terminate the labeling reaction. The cells were left on ice for 15 min and lysed as described above. Biotin-labeled cell surface proteins were isolated with NeutrAvidin beads (Thermo Fisher, 89,881) and examined by western blotting with an anti-V5 (1:5000, Themo Fisher, R961–25) or FLAG antibody (1:2000, Sigma, A8592). To analyze CD320 fragments that were shed or secreted in the conditioned media, immunoprecipitation was done with an anti-FLAG (1:10,000, HuaBio, M1403–2) or V5 (1:5000, Themo Fisher, R960–25) antibody at 4 °C. After 16 h, protein A-Sepharose beads (Thermo Fisher, 101,042) were added to pull-down the antibody-coupled proteins for SDS-PAGE and western blotting examination.

To validate proteins on the cell surface, trypsin digestion was carried out. The transfected cells expressing CD320 were treated with 0.05% Trypsin-EDTA (Gibco, 25,300) on ice. At different time points, DMEM with 10% FBS was added to the cells to terminate trypsin activity. The cells were collected and transferred to fresh tubes, washed with PBS, and lysed with the buffer as described above. Proteins in the lysates were analyzed by SDS-PAGE and western blotting.

### Effects of BFA, monensin, and Ben-gal

To assess CD320 in subcellular compartments, the transfected HEK293 cells expressing CD320 or corin (control) were treated with BFA (0.1–2 μm) (Sigma, 203,729) and monensin (0.05–2 μm) (Sigma, 475,897) in dimethylsulfoxide (DMSO) or the O-glycosylation inhibitor Ben-gal (8 mM) (Sigma, B4894) in DMEM. As controls, the cells were treated with vehicles (DMSO or DMEM). After 24 h at 37 °C, the cells were washed with PBS and lysed. CD320 and corin bands in the lysates were analyzed by SDS-PAGE and western blotting under reducing and non-reducing conditions.

### LC-MS analysis

HEK293 cells were transfected with the plasmid expressing FLAG-tagged WT CD320 or a vector control. After 24 h at 37 °C, the conditioned media were collected. Immunoprecipitation was done with an anti-FLAG antibody coupled with protein A-Sepharose beads (Sangon Biotech, C600957). After washing, proteins were eluted from the beads with the non-reducing loading buffer and separated by SDS-PAGE. The gel was silver-stained. Gel sections were cut at the ∼310 to 350 kDa position, destained, and digested with trypsin (12.5 μg/ml) at 37 °C overnight. Peptides were extracted twice with 50% acetonitrile and 0.1% (v/v) trifluoroacetic acid, dried, and reconstituted in 0.1% formic acid. LC-MS was done at Shanghai Huazhi Biotechnology, China. Briefly, the samples were run using Bruker timsTOF Pro2 equipment (Bruker Daltonics) with a column (25 cm × 75 μm, IonOpticks) at 60 °C and a flow rate of 200 nl/min. Data were acquired in positive mode (m/z 350–1700) with parallel accumulation serial fragmentation mode. Raw data were analyzed by PEAKS Online. Differential proteins were analyzed in RStudio and presented in a volcano plot showing log_2_ fold change and −log_10_ *p* values.

### Cellular uptake of vitamin B_12_

HepG2 cells were transfected with a vector or plasmids expressing CD320 WT and the deletion mutants and cultured in DMEM containing 2% FBS and 500 nM vitamin B_12_ (MedChemExpress, HY-B0315). After 36 h at 37 °C, the cells were detached after trypsin (0.25%, KeyGEN Biotech, KGL2101–100) treatment and lysed by sonication. Proteins in the lysates were quantified by a bicinchoninic acid assay. Vitamin B_12_ levels in the lysates were measured by an ELISA kit (Shanghai Yansheng Industrial, YS-S964804) according to the manufacturer’s instructions.

### Cycloheximide (CHX) treatment

To analyze CD320 intracellular trafficking, HEK293 cells expressing CD320 WT and the ΔE88 variant were treated with CHX (100 μg/ml, Sigma, 239,765) at 37 °C to block protein synthesis. At various times up to 12 h, the cells were washed and lysed as described above. Proteins in lysates were analyzed by SDS-PAGE and western blotting under reducing conditions. Levels of CD320 bands on Western blots were quantified by densitometry.

### 3D model prediction by AlphaFold3

The artificial intelligence-based AlphaFold3 software was used to predict the 3D structures of the human CD320 oligomer. Five copies of the human CD320 extracellular protein sequence (residues 36–230) were submitted to the AlphaFold3 website (https://www.alphafoldserver.com) (41). A 3D model of CD320 pentamer was generated and downloaded. The structure file was imported into PyMOL software for visualization and analysis.

### Statistical analyses

Data were examined using Prism software (V9.0, GraphPad Prism). Data normal distribution of was verified using Shapiro–Wilk test. Student’s *t* test was used to compare data between two groups. One-way ANOVA followed by Bonferroni’s *post hoc* analysis was used to examine data from three or more groups. A *p* value of <0.05 is considered significant. Data are presented as mean ± SD

## Data availability

All primary data are available upon request.

## Supporting information

This article has supporting information.

## Conflict of interest

The authors declare no conflict of interest with the contents of this article.
