# Supplementary material for: Transcobalamin receptor CD320, responsible for vitamin B12 cellular uptake, is present on the cell surface as a homo-oligomer
Source: J Biol Chem. 2026 Jul 16;302(8):113286. doi: 10.1016/j.jbc.2026.113286 (PMC13382787; doi:10.1016/j.jbc.2026.113286)
Supplement: Supplementary Material [file mmc1.pdf]

Supporting Information

Transcobalamin receptor CD320, responsible for vitamin B_{12} cellular uptake, is present on the cell surface as a homo-oligomer

Wenjun Guo, Renping Qiu, Xiaotong Zhao, Tiantian Zhou, Meng Liu, Ningzheng Dong, and Qingyu Wu

Supporting Figure List:

Figure S1. A 3D pentamer model of CD320 predicted by AlphaFold3

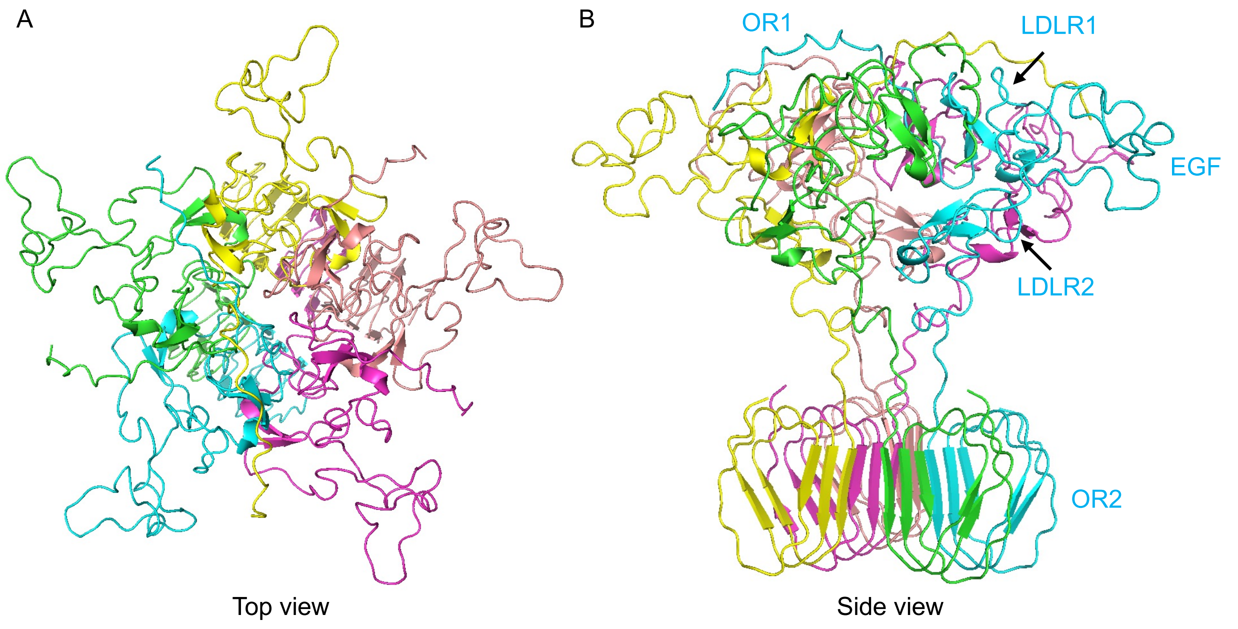

Figure S1. A 3D pentamer model of CD320 predicted by AlphaFold3. *A* and *B*, Five copies of the human CD320 extracellular protein sequence (residues 36-230) were analyzed by AlphaFold3 software. The predicted 3D pentamer structure of CD320 is presented in top (A) and side (B) views. Each CD320 monomer is shown in a separate color. On the side view (B), the OR1, LDLR1, EGF, LDLR2 and OR2 domains of one CD320 monomer (blue) are indicated.
